# Data of high performance precast external walls for warm climate^☆^

**DOI:** 10.1016/j.dib.2015.07.004

**Published:** 2015-07-15

**Authors:** Cristina Baglivo, Paolo Maria Congedo

**Affiliations:** Department of Engineering for Innovation, University of Salento, 73100 Lecce, Italy

## Abstract

The data given in the following paper are related to input and output information of the paper entitled Design method of high performance precast external walls for warm climate by multi-objective optimization analysis by Baglivo et al. [1].

Previous studies demonstrate that the superficial mass and the internal areal heat capacity are necessary to reach the best performances for the envelope of the Zero Energy Buildings located in a warm climate [2–4]. The results show that it is possible to achieve high performance precast walls also with light and ultra-thin solutions.

A multi-criteria optimization has been performed in terms of steady and dynamic thermal behavior, eco sustainability score and costs. The modeFRONTIER optimization tool, with the use of computational procedures developed in Matlab, has been used to assess the thermal dynamics of building components.

A large set of the best configurations of precast external walls for warm climate with their physical and thermal properties have been reported in the data article.

**Table:** Specifications Table

Subject area	*Civil engineering,*
More specific subject area	*Dynamic thermal performance of external walls*
Type of data	*Tables*
How data was acquired	*Puglia price list, technical data sheet, Analyzed and processed output data*
Data format	*xls*
Experimental factors	*No pretreatment of samples was performed*
Experimental features	*A methodology to design several precast walls for warm climate by the use of eco-friendly materials is shown.*
	*The building materials have been proposed considering the optimum balance between the energy performance, the environmental impact, and cost.*
Data source location	*Data is given within this article.*
Data accessibility	*Data is given within this article.*

*Value of the data*

An effective methodology is applied to get high efficiency precast walls for Mediterranean climate for several thicknesses. Steady and dynamic performances of each wall have been shown. The costs and the eco-friendly score are evaluated for precast walls classified according to the total thicknesses (thick, thin and ultra-thin precast walls).

## 1 Data, experimental design, materials and methods

The methodology of this research [1] has been carried out by the following main steps:

1) Implementation of a database consisting of several building materials available on the market;
2) Elaboration of a script for the calculation of thermal-dynamic properties of package walls, according with the UNI EN ISO 13786:2008 (Thermal performance of building components, Dynamic Thermal Characteristics, Calculation Methods)[5], and for the evaluation of eco-friendly score and costs;
3) Definition of performance values, the objectives and constraints required to obtain high performance precast external walls for warm climate;
4) Analysis of optimal solutions, i.e. the best compromise among the various objectives and all the constraints imposed on the problem.

The selection of building materials is considered as a multi-objective decision; the designer can choose the appropriate solution for his work from a number of possible configurations of the external wall.

The modeFRONTIER optimization tool, with the use of computational procedures developed in Matlab, has been used in order to calculate the thermal dynamics performances of building components. The optimization has been carried out in terms of steady thermal transmittance, periodic thermal transmittance, decrement factor, time shift, areal heat capacity, thermal admittance, surface mass, small thickness, eco sustainability score, light-weight and costs. The superficial mass and the internal areal heat capacity are necessary to reach the best performances for the envelope of the Zero Energy Buildings located in a warm climate [2–4].

The analysis of the results suggests that, to obtain high performance walls for warm climates, it is strongly required mass in the inner side that is able to increase the thermal inertia and gives to the wall the ability to store and discharge with a certain time shift the flow of the heat. For this reason, concrete is disposed in the first layer (internal side).

Several authors [6–8] show the benefits of thermal inertia and evaluate how the walls thermal properties influence a building׳s energy performance. In particular, by the right choice of isolation and an optimal thickness is possible to reduce the heat flow that crosses the multi-layered wall and the energy consumption of the entire building [9].

The results show that it is possible to reach highly performing behavior also with limited thicknesses, less than 40 cm, using ecological materials available today on the market, and achieve high thermal dynamic performances for hot climates.

This study, defining a method for the choice of construction materials sequences in the precast external walls, contributes to the reduction of energy consumption in a building [10–12] and supports the design of Nearly zero-energy buildings.
